# *N*-(3-Chloro­phen­yl)maleimide

**DOI:** 10.1107/S1600536808011604

**Published:** 2008-04-30

**Authors:** Rodolfo Moreno-Fuquen, Zulay Pardo-Botero, Javier Ellena

**Affiliations:** a Departamento de Química - Facultad de Ciencias, Universidad del Valle, Apartado 25360, Santiago de Cali, Colombia; b Facultad de Ciencias Químicas, Universidad Complutense de Madrid, Avenida Complutense s/n, 28040 Madrid, Spain; c Instituto de Física, Universidade de São Paulo, São Carlos, Brazil.

## Abstract

The title compound, C_10_H_6_ClNO_2_, has a dihedral angle of 46.46 (5)° between the benzene and maleimide rings. A short inter­molecular halogen–oxygen contact is observed, with a Cl⋯O distance of 3.0966 (13) Å. Both CO groups are involved in two C—H⋯O inter­actions, which gives rise to sheets parallel to (100). In addition, these sheets exhibit a π–π stacking inter­action between the benzene and maleimide rings [mean inter­planar distance of 3.337 (3) Å].

## Related literature

For related literature, see: Etter (1990 ▶); Howell & Zhang (2006 ▶); Metrangolo & Resnati (2001 ▶); Miller *et al.* (2000 ▶, 2001 ▶); Moreno-Fuquen *et al.* (2006 ▶); Sureshan *et al.* (2001 ▶).
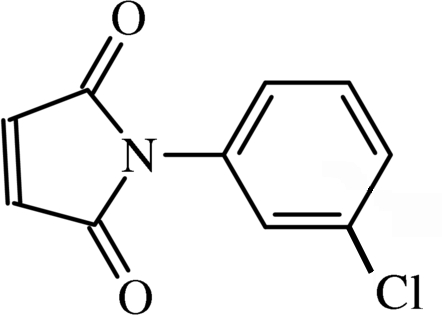


## Experimental

### 

#### Crystal data

C_10_H_6_ClNO_2_
*M* *_r_* = 207.61
Monoclinic, 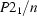

*a* = 7.3434 (3) Å
*b* = 11.9458 (5) Å
*c* = 10.3044 (4) Å
β = 101.121 (2)°
*V* = 886.96 (6) Å^3^
*Z* = 4
Mo *K*α radiation
μ = 0.40 mm^−1^
*T* = 291 (2) K
0.18 × 0.10 × 0.04 mm

#### Data collection

Bruker–Nonius KappaCCD diffractometer
Absorption correction: none
4426 measured reflections
2042 independent reflections
1680 reflections with *I* > 2σ(*I*)
*R*_int_ = 0.036

#### Refinement

*R*[*F* ^2^ > 2σ(*F* ^2^)] = 0.038
*wR*(*F* ^2^) = 0.101
*S* = 1.08
2042 reflections
128 parameters
H-atom parameters constrained
Δρ_max_ = 0.25 e Å^−3^
Δρ_min_ = −0.39 e Å^−3^

### 

Data collection: *DENZO* (Otwinowski & Minor, 1997 ▶) and *COLLECT* (Nonius, 2000 ▶); cell refinement: *DENZO* and *COLLECT*; data reduction: *DENZO* and *COLLECT*; program(s) used to solve structure: *SHELXS97* (Sheldrick, 2008 ▶); program(s) used to refine structure: *SHELXL97* (Sheldrick, 2008 ▶); molecular graphics: *ORTEP-3 for Windows* (Farrugia, 1997 ▶); software used to prepare material for publication: *PARST95* (Nardelli, 1995 ▶).

## Supplementary Material

Crystal structure: contains datablocks I, global. DOI: 10.1107/S1600536808011604/fj2114sup1.cif

Structure factors: contains datablocks I. DOI: 10.1107/S1600536808011604/fj2114Isup2.hkl

Additional supplementary materials:  crystallographic information; 3D view; checkCIF report

## Figures and Tables

**Table 1:** Hydrogen-bond geometry (Å, °)

*D*—H⋯*A*	*D*—H	H⋯*A*	*D*⋯*A*	*D*—H⋯*A*
C3—H3⋯O2^i^	0.93	2.53	3.455 (2)	170
C8—H8⋯O2^ii^	0.93	2.71	3.513 (2)	146
C8—H8⋯O1^iii^	0.93	2.59	3.256 (2)	129
C2—H2⋯O1^iv^	0.93	2.72	3.308 (2)	122

Symmetry codes: (i) 
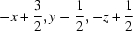
; (ii) 
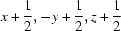
; (iii) 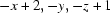; (iv) 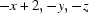.

**Fig. 1.**
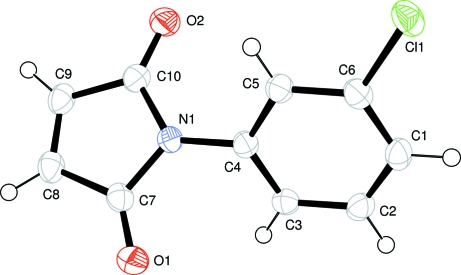
An ORTEP-3 (Farrugia, 1997) plot of (I), with the atomic labelling scheme. The shapes of the ellipsoids correspond to 50% probability contours of atomic displacement.

**Fig. 2.**
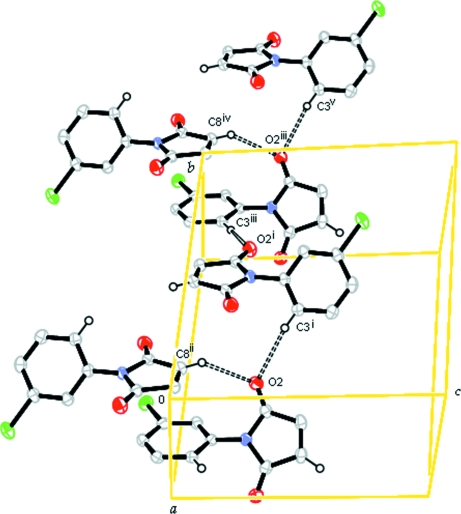
Part of the crystal structure of (I) showing the formation of the C(12) and C(13) chains along [010]. Symmetry codes: (i) 3/2 - x, 1/2 + y, 1/2 - z; (ii) -1/2 + x, 1/2 - y, -1/2 + z; (iii) x, 1 + y, z; (iv) -1/2 + x, 1/2 - y, -1/2 + z; (v) 3/2 - x, 3/2 + y, 1/2 - z. For the sake of clarity, the H atoms not involved in the motif shown have been omitted.

**Fig. 3.**
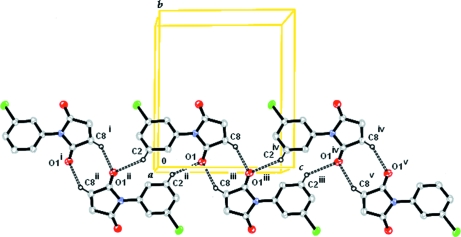
Part of the crystal structure of (I) showing the formation of a sheet of R22(8) and R22(14) centrosymmetric rings parallel to (100) generated by the C—H···O hydrogen bonds. Symmetry codes: (i) x, y, 1 - z; (ii) -x, -y, -z; (iii) -x, -y, 1 - z; (iv) x, y, 1 + z; (v) -x, -y, 2 - z. For the sake of clarity, the H atoms not involved in the motif have been omitted.

**Fig. 4.**
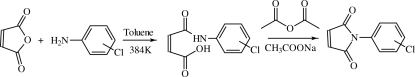
The formation of the title compound.

## supplementary crystallographic information

### Comment

Due to the interest created by the N-substituted maleimides in free radical polymerization process upon exposure to light (Howell & Zhang, 2006), the synthesis and study of the crystal structure of *N*-(*m*-chlorophenylmaleimide) (I) was undertaken. *N*-(*m*-nitrophenylmaleimide) (3NPMI) (Moreno-Fuquen *et al.*, 2006) and *N*-(*o*-chlorophenyl) maleimide (2ClPMI) (Miller *et al.*, 2001) systems can be taken as a reference systems to compare with the structural characteristics of (I). Perspective view of (I), showing the atomic numbering scheme, is given in Fig. 1. Photochemical properties of arylmaleimide systems have shown that they depend on the value of the dihedral angle between the benzene and imidic rings (Miller *et al.*, 2000). This angle is 56.2 (1)° and 52.9 (1)° for 3NPMI, 66.10 (4) ° for 2ClPMI, and 46.46 (5)° for (I). The molecules of (I) are linked into sheets by a combination of C—H···O hydrogen bonds (Nardelli, 1995) (Table 1). Indeed, the atoms C3^i^ in the molecule at (3/2 - *x*, 1/2 + *y*, 1/2 - *z*) and C8^ii^ in the molecule at (-1/2 + *x*, 1/2 - *y*, -1/2 + *z*) act as hydrogen-bond donors to maleimidic O2 atom in the molecule at (*x*, *y*, *z*), so generating by 2_1_ screw axis a C(6) chain (Etter, 1990), which is running parallel to [010] direction (Fig. 2, supp. material). Within the asymmetric unit the atom C2 at (*x*, *y*, *z*) acts as hydrogen bond donor to maleimidic O1^iii^ in the molecule at (2 - *x*, -*y*, -*z*), so forming by translation a *R*_2_^2^(14) centrosymmetric rings (Etter, 1990); in addition, atom C8 at (*x*, *y*, *z*) acts as a hydrogen bond donor to maleimidic O1^ii^ in the molecule at (2 - *x*, -*y*, 1 - *z*), so generating by translation a *R*_2_^2^(8) centrosymmetric rings. Both rings are running along [001] direction (Fig.3, supp. material). In addition, (I) exhibits an aromatic π···π stacking interactions between benzene and maleimide rings with a mean interplanar distance of 3.337 (3) Å. The halogen-oxygen interaction is recognized as a strong driving force in formation of molecular crystals (Sureshan *et al.*, 2001). (I) shows a short Cl···O intermolecular contact, disposed about an inversion centre. The Cl1···O2, shows a distance of 3.0966 (13) Å, [O2 with symmetry 2 - *x*, 1 - *y*, -*z*] and this contact is shorter than the sum of their van der Waalś radii (3.27 Å, Metrangolo & Resnati, 2001). In (I), the angle of the oxygen O2 relative to the C6—Cl bond shows a slight deviation from linearity with a value of 174.31 (6)° and the angle of the chlorine atom relative to the C10—O2 bond shows a value of 136.96 (11)°, suggesting strong halogen bonding. This could also prevent a larger rotation between the planes of (I). The title system does not have enough influence on the processes of polymerization because the dihedral angle between their rings possess a low value with respect to other systems with substituents in the position *ortho* (Miller *et al.*, 2000).

### Experimental

Reagents and solvents for the synthesis were from Aldrich Chemical Co. and they were used without additional purification. Column chromatography was performed using silica gel H60 to purify the intermediates and final products. Thin layer chromatography (TLC) was used to confirm the structure of the individual compounds.

### Refinement

The space group P 2_1_/n for (I) was uniquely assigned from the systematic absences. All H-atoms were located from difference maps and then treated as riding atoms [C—H= 0.93Å and *U*_iso_(H)= 1.2*U*_eq_(C)].

### Crystal data

**Table:**

C_10_H_6_ClNO_2_	*F*_000_ = 424
*M**_r_* = 207.61	*D*_x_ = 1.550 Mg m^−^^3^
Monoclinic, *P*2_1_/*n*	Melting point: 364(1) K
Hall symbol: -P 2yn	Mo *K*α radiation λ = 0.71073 Å
*a* = 7.3434 (3) Å	Cell parameters from 4426 reflections
*b* = 11.9458 (5) Å	θ = 2.9–27.5º
*c* = 10.3044 (4) Å	µ = 0.40 mm^−^^1^
β = 101.121 (2)º	*T* = 291 (2) K
*V* = 886.96 (6) Å^3^	Needle, colorless
*Z* = 4	0.18 × 0.10 × 0.04 mm

### Data collection

**Table:**

Bruker–Nonius KappaCCD diffractometer	1680 reflections with *I* > 2σ(*I*)
Radiation source: fine-focus sealed tube	*R*_int_ = 0.036
Monochromator: graphite	θ_max_ = 27.5º
φ and ω scans	θ_min_ = 2.9º
Absorption correction: none	*h* = −9→8
4426 measured reflections	*k* = −15→15
2042 independent reflections	*l* = −13→13

### Refinement

**Table:**

Refinement on *F*^2^	Hydrogen site location: inferred from neighbouring sites
Least-squares matrix: full	H-atom parameters constrained
*R*[*F*^2^ > 2σ(*F*^2^)] = 0.038	*w* = 1/[σ^2^(*F*_o_^2^) + (0.0496*P*)^2^ + 0.2501*P*] where *P* = (*F*_o_^2^ + 2*F*_c_^2^)/3
*wR*(*F*^2^) = 0.101	(Δ/σ)_max_ < 0.001
*S* = 1.09	Δρ_max_ = 0.25 e Å^−^^3^
2042 reflections	Δρ_min_ = −0.39 e Å^−^^3^
128 parameters	Extinction correction: SHELXL97 (Sheldrick, 2008), Fc^*^=kFc[1+0.001xFc^2^λ^3^/sin(2θ)]^-1/4^
Primary atom site location: structure-invariant direct methods	Extinction coefficient: 0.031 (5)
Secondary atom site location: difference Fourier map	

### Special details

**Table:**

Geometry. All e.s.d.'s (except the e.s.d. in the dihedral angle between two l.s. planes) are estimated using the full covariance matrix. The cell e.s.d.'s are taken into account individually in the estimation of e.s.d.'s in distances, angles and torsion angles; correlations between e.s.d.'s in cell parameters are only used when they are defined by crystal symmetry. An approximate (isotropic) treatment of cell e.s.d.'s is used for estimating e.s.d.'s involving l.s. planes.

### Fractional atomic coordinates and isotropic or equivalent isotropic displacement parameters (Å^2^)

**Table:**

	*x*	*y*	*z*	*U*_iso_*/*U*_eq_	
Cl1	1.04083 (6)	0.39876 (4)	−0.16126 (4)	0.03549 (17)	
O1	1.08785 (18)	0.03863 (10)	0.33200 (12)	0.0348 (3)	
O2	0.83189 (18)	0.38885 (10)	0.29127 (12)	0.0348 (3)	
N1	0.95758 (18)	0.21271 (11)	0.27188 (13)	0.0253 (3)	
C1	0.8978 (2)	0.19208 (15)	−0.14130 (16)	0.0309 (4)	
H1	0.8853	0.1877	−0.2327	0.037*	
C2	0.8435 (2)	0.10389 (13)	−0.07035 (17)	0.0300 (4)	
H2	0.7944	0.0396	−0.1148	0.036*	
C3	0.8613 (2)	0.10986 (13)	0.06635 (16)	0.0267 (4)	
H3	0.8224	0.0507	0.1131	0.032*	
C4	0.9379 (2)	0.20552 (13)	0.13225 (15)	0.0245 (3)	
C5	0.9937 (2)	0.29538 (13)	0.06274 (15)	0.0267 (4)	
H5	1.0448	0.3594	0.1067	0.032*	
C6	0.9710 (2)	0.28685 (14)	−0.07360 (16)	0.0285 (4)	
C7	1.0295 (2)	0.12740 (14)	0.36145 (16)	0.0280 (4)	
C8	1.0201 (2)	0.17113 (15)	0.49571 (16)	0.0321 (4)	
H8	1.0594	0.1332	0.5750	0.038*	
C9	0.9469 (2)	0.27274 (14)	0.48355 (16)	0.0320 (4)	
H9	0.9263	0.3176	0.5530	0.038*	
C10	0.9026 (2)	0.30376 (13)	0.34085 (16)	0.0276 (4)	

### Atomic displacement parameters (Å^2^)

**Table:**

	*U*^11^	*U*^22^	*U*^33^	*U*^12^	*U*^13^	*U*^23^
Cl1	0.0406 (3)	0.0386 (3)	0.0306 (3)	0.00442 (17)	0.01531 (18)	0.01000 (17)
O1	0.0433 (7)	0.0275 (6)	0.0336 (7)	0.0019 (5)	0.0076 (5)	0.0034 (5)
O2	0.0438 (7)	0.0307 (7)	0.0318 (7)	0.0066 (5)	0.0122 (5)	0.0004 (5)
N1	0.0308 (7)	0.0245 (7)	0.0215 (7)	−0.0005 (5)	0.0071 (5)	0.0015 (5)
C1	0.0321 (8)	0.0380 (9)	0.0236 (8)	0.0098 (7)	0.0074 (6)	−0.0012 (7)
C2	0.0310 (8)	0.0294 (8)	0.0290 (8)	0.0061 (6)	0.0047 (7)	−0.0048 (6)
C3	0.0277 (8)	0.0254 (8)	0.0270 (8)	0.0030 (6)	0.0056 (6)	0.0007 (6)
C4	0.0245 (7)	0.0270 (8)	0.0228 (7)	0.0039 (6)	0.0063 (6)	0.0005 (6)
C5	0.0284 (8)	0.0280 (8)	0.0251 (8)	0.0015 (6)	0.0083 (6)	−0.0001 (6)
C6	0.0286 (8)	0.0314 (8)	0.0277 (8)	0.0064 (6)	0.0110 (6)	0.0043 (6)
C7	0.0295 (8)	0.0278 (8)	0.0265 (8)	−0.0057 (6)	0.0050 (6)	0.0039 (6)
C8	0.0376 (9)	0.0355 (9)	0.0237 (8)	−0.0080 (7)	0.0073 (7)	0.0024 (7)
C9	0.0389 (9)	0.0351 (9)	0.0240 (8)	−0.0076 (7)	0.0109 (7)	−0.0021 (7)
C10	0.0290 (8)	0.0285 (8)	0.0270 (8)	−0.0032 (6)	0.0093 (6)	−0.0012 (6)

### Geometric parameters (Å, °)

**Table:**

Cl1—C6	1.7450 (17)	C3—C4	1.392 (2)
O1—C7	1.204 (2)	C3—H3	0.9300
O2—C10	1.209 (2)	C4—C5	1.395 (2)
N1—C10	1.401 (2)	C5—C6	1.386 (2)
N1—C7	1.408 (2)	C5—H5	0.9300
N1—C4	1.421 (2)	C7—C8	1.493 (2)
C1—C6	1.383 (2)	C8—C9	1.324 (3)
C1—C2	1.384 (2)	C8—H8	0.9300
C1—H1	0.9300	C9—C10	1.490 (2)
C2—C3	1.391 (2)	C9—H9	0.9300
C2—H2	0.9300		
			
C10—N1—C7	109.76 (13)	C4—C5—H5	120.9
C10—N1—C4	125.30 (13)	C1—C6—C5	122.04 (15)
C7—N1—C4	124.90 (13)	C1—C6—Cl1	119.38 (13)
C6—C1—C2	118.70 (15)	C5—C6—Cl1	118.57 (13)
C6—C1—H1	120.6	O1—C7—N1	125.39 (15)
C2—C1—H1	120.6	O1—C7—C8	128.61 (16)
C1—C2—C3	121.03 (15)	N1—C7—C8	106.00 (14)
C1—C2—H2	119.5	C9—C8—C7	108.89 (15)
C3—C2—H2	119.5	C9—C8—H8	125.6
C2—C3—C4	119.09 (15)	C7—C8—H8	125.6
C2—C3—H3	120.5	C8—C9—C10	109.20 (15)
C4—C3—H3	120.5	C8—C9—H9	125.4
C3—C4—C5	120.84 (15)	C10—C9—H9	125.4
C3—C4—N1	119.76 (14)	O2—C10—N1	125.51 (15)
C5—C4—N1	119.40 (14)	O2—C10—C9	128.34 (15)
C6—C5—C4	118.28 (15)	N1—C10—C9	106.14 (14)
C6—C5—H5	120.9		
			
C6—C1—C2—C3	0.2 (2)	C10—N1—C7—O1	179.89 (16)
C1—C2—C3—C4	−1.1 (2)	C4—N1—C7—O1	1.9 (3)
C2—C3—C4—C5	1.1 (2)	C10—N1—C7—C8	−0.97 (17)
C2—C3—C4—N1	−179.78 (14)	C4—N1—C7—C8	−178.92 (14)
C10—N1—C4—C3	−131.50 (16)	O1—C7—C8—C9	179.73 (17)
C7—N1—C4—C3	46.1 (2)	N1—C7—C8—C9	0.62 (18)
C10—N1—C4—C5	47.7 (2)	C7—C8—C9—C10	−0.05 (19)
C7—N1—C4—C5	−134.69 (16)	C7—N1—C10—O2	−178.35 (16)
C3—C4—C5—C6	−0.1 (2)	C4—N1—C10—O2	−0.4 (3)
N1—C4—C5—C6	−179.23 (14)	C7—N1—C10—C9	0.94 (17)
C2—C1—C6—C5	0.9 (2)	C4—N1—C10—C9	178.88 (14)
C2—C1—C6—Cl1	179.82 (12)	C8—C9—C10—O2	178.72 (17)
C4—C5—C6—C1	−0.9 (2)	C8—C9—C10—N1	−0.54 (19)
C4—C5—C6—Cl1	−179.88 (11)		

### Hydrogen-bond geometry (Å, °)

**Table:**

*D*—H···*A*	*D*—H	H···*A*	*D*···*A*	*D*—H···*A*
C3—H3···O2^i^	0.93	2.53	3.455 (2)	170
C8—H8···O2^ii^	0.93	2.71	3.513 (2)	146
C8—H8···O1^iii^	0.93	2.59	3.256 (2)	129
C2—H2···O1^iv^	0.93	2.72	3.308 (2)	122

Symmetry codes: (i) −*x*+3/2, *y*−1/2, −*z*+1/2; (ii) *x*+1/2, −*y*+1/2, *z*+1/2; (iii) −*x*+2, −*y*, −*z*+1; (iv) −*x*+2, −*y*, −*z*.
